# Laboratory and Field Bioassays of Arthropod Pathogenic Fungi Application for the Control of the Hazelnut Big Bud Mite, *Phytoptus avellanae s. l.*

**DOI:** 10.3390/insects16111182

**Published:** 2025-11-20

**Authors:** Domenico Valenzano, Ilaria Laterza, Mario Contarini, Stefano Speranza, Roberto Masturzi, Eustachio Tarasco, Enrico de Lillo

**Affiliations:** 1 Dipartimento di Scienze Agrarie e Forestali (DAFNE), University of Tuscia, Via San Camillo de Lellis snc, 01100 Viterbo, Italy; domenico.valenzano@unitus.it (D.V.); contarini@unitus.it (M.C.); speranza@unitus.it (S.S.); roberto.masturzi@unitus.it (R.M.); 2 Dipartimento di Scienze del Suolo, della Pianta e degli Alimenti (DiSSPA), University of Bari Aldo Moro, Via Amendola, 165/a, 70126 Bari, Italy; ilaria.laterza@uniba.it (I.L.); eustachio.tarasco@uniba.it (E.T.)

**Keywords:** Eriophyoidea, gall-making mites, *Corylus avellana*, microbiological control, *Beauveria bassiana*, *Akanthomyces muscarius*, laboratory assays, field trial

## Abstract

*Phytoptus avellanae* is a key hazelnut pest, causing the swelling of mixed buds during the autumn–winter period and a compromised nut yield. This study aimed to evaluate the effectiveness of commercial biopesticides based on *Beauveria bassiana* and *Akanthomyces muscarius* in controlling this pest. Specific laboratory and field protocols were developed. In the laboratory, bud galls were disinfected, dissected, immersed for 30 s in a water suspension of the products and in water (control), dried in a laminar flow hood, incubated for 7 days and inspected to assess mite mortality. Intermediate observations were also recorded. Significantly higher mortality was recorded in the treatment with *B. bassiana*. Infection with *B. bassiana* was confirmed by a culture in medium and slide-mounting of infected mites. The orchard assay was performed by immersing buds and nuts in the fungal suspensions applied in the laboratory without separating any organ from the plant. Two weeks after application, the treated plant organs were removed and examined to assess mite mortality. The organs treated with *B. bassiana* showed higher mite mortality than the control for both buds and nuts. Treatments with arthropod pathogenic fungi appear to be promising for inclusion in the control strategy of *P. avellanae*.

## Simple Summary

Hazelnut is becoming increasingly important economically in the Mediterranean basin: specifically, in Turkey and Italy, where most of the world’s hazelnut production is concentrated. *Phytoptus avellanae* is increasingly involved in severe infestations that cause plant growth disorders and a reduction in hazelnut yield. Recently, it has been proposed that a free-living form of this species (still unnamed) could be involved in injuries to other organs of the hazelnut. This study explores the application of low-impact control strategies against *P. avellanae s. l.*, through the application of arthropod pathogenic fungi (APF). Laboratory and field assays were conducted on mites using commercial fungal control products at the doses indicated on the label. The laboratory assays were repeated in the winter of 2023–2024, the winter of 2024–2025, and the spring of 2025. They took place in two different laboratories, under the same experimental conditions. In 2024, a field assay was conducted with the same fungi in a commercial hazelnut orchard. In both types of assays, the application of *Beauveria bassiana* and *Akanthomyces muscarius* demonstrated promising efficacy in controlling the pest, supporting their potential introduction into the integrated management of *P. avellanae*.

## 1. Introduction

The cultivation of hazelnut (*Corylus avellana* L.) has considerable economic importance in several regions worldwide, including Italy, where it contributes substantially to the agricultural sector. Hazelnut production is threatened by various pests: in particular, eriophyoid mites, which can cause considerable damage to trees and nuts. The hazelnut bud mite, *Phytoptus avellanae* Nalepa (Eriophyoidea: Phytoptidae, Phytoptinae), stimulates bud enlargement—commonly referred to as “big bud galls”—which compromises the normal growth of leaves, flowers and fruits, resulting in reduced nut yield and quality [1]. Quite recently, a cryptic species was separated by *P. avellanae sensu stricto*, based on the mitochondrial and nuclear DNA [2]. This species, which is still unnamed and included in the *P. avellanae sensu lato* complex, is considered to be free-living on the surfaces of green plant organs, but it remains poorly characterized in terms of bio-ecology, morphology, and the type of injuries it causes to hazelnut [3]. The gall-making species overwinters inside the galls [3], which protect it from adverse conditions, while also making control measures more challenging. The free-living species is also supposed to overwinter inside buds [2]. During the spring bud break, the gall-making species move to the new buds, whereas the free-living species move to the green organ surfaces.

The main strategy for controlling eriophyoids relies on the application of acaricides and other pesticides. Over the last two decades, there has been a significant reduction in the variety of authorized synthetic molecules with pesticide activity, especially in the European Union. Plant protection products based on sulfur, copper oxychloride, oil, fatty acids, unsaturated potassium salts and nettle extract are currently only authorized in Italy for the control of *P. avellanae sensu stricto* (interrogation of https://fitogest.imagelinenetwork.com/ accessed on 31 August 2025). The reduced availability of acaricides, along with the increasing concerns about the environmental impact of synthetic products, the development of mite resistance, and the potential side effects on beneficial fauna, have stimulated interest in alternative strategies to chemical control. Arthropod pathogenic fungi (APF) are promising agents for the microbiological control of insects and mites [4]. These fungi infect the host through direct contact, leading to its death without the need for ingestion of spores/conidia [5], which could be challenging for plant feeder mites with stylet-like mouthparts like eriophyoids [6]. APF have been studied for the management of some species of mites, as well as numerous insects. *Beauveria bassiana* (Bals.) Vuill., *Metarhizium anisopliae* (Metsch.) Sorokin, *Cordyceps tenuipes* (Peck) Kepler, Shrestha and Spatafora (Hypocreales, Clavicipitaceae and Cordycipitaceae) were observed to be effective against spider mites [5,7,8] and eriophyoid mites [5,9]. Recently, a strain of *Akanthomyces muscarius* (Petch) Spatafora, Kepler and Shrestha, has also been characterized from hazelnut buds infested by *P. avellanae* [10].

Despite their potential as sustainable control agents, the extremely small size of eriophyoid mites, their hidden lifestyle and their strict dependence on young host plant organs [11] have hindered research into the use of APF for their control, and specifically for the gall-making and refuge-seeking ecological classes. Little literature has been published on this topic and most studies and protocols evaluating the effectiveness of APF have focused mostly on species living on exposed plant surface organs [12,13,14,15,16]. Obviously, the classified free-living species are easier to manage in the laboratory together with their host plants or parts of them, in contrast with the gall-making species, which are much more demanding for ecological factors. *Phytoptus avellanae sensu stricto* can be much more easily reached by APF during its spring migration from the big buds to the new buds or green organs. The migration time of the gall-making species population is reported to last about one month and a half, but the exposure of the single mite is reduced to a few hours or days. Therefore, the bio-ethology of this mite does not allow for planning laboratory trials on stems during migration. Vice versa, the efficiency of the APF on this species can be evaluated on the mites that are available inside the bud galls for months.

Kanagaratnam et al. [17] investigated the APF–mite relationship in the gall-making species *Cecidophyopsis ribis* (Westwood) (Eriophyoidea, Eriophyidae, Cecidophyinae, Cecidophyini). This mite species induces terminal or lateral buds of blackcurrants, *Ribes nigrum* L., to become swollen and rounded up to about 1 cm across. These authors succeeded in keeping the mites alive in the galls long enough for naturally occurring APF to develop inside them, thereby linking fungal development to mite mortality. Abramishvili and Burjanadze [18] provided a short but not exhaustive description of the protocols applied with a native fungal strain (Bb-024) of *B. bassiana* in the laboratory and semi-field efficiency assays against *P. avellanae*. However, the positive outcomes of the experiments illustrated that both gall-making species provided a strong rationale for further research employing commercial APF strains in order to optimize their application. Environmental conditions, application methods and fungal strain selection require careful evaluation to improve the efficacy and sustainability of these biopesticides. In addition, no APF are authorized against *P. avellanae* and trials are not reported in the literature, showing a knowledge gap which needs to be addressed.

Therefore, this study aims to establish a protocol for assessing the potential application of APF in laboratory conditions and in the field, as well as for verifying the efficacy of two APF available on the market for the control of *P. avellanae*.

## 2. Materials and Methods

### 2.1. Arthropod Pathogenic Fungi

Based on the previous studies [10,18,19], two commercial APF were selected for the comparison: *B. bassiana* strain ATCC 74040 (Naturalis^®^, oil suspension, by BIOGARD^®^, Grassobbio, Italy) (indicated as NAT in the text) and the *Akanthomyces muscarius* strain, Ve6 (Mycotal^®^, powdered formulation, by KOPPERT Italia, Bussolengo, Italy) (indicated as MYC in the text). Each formulation was suspended in sterile water with 0.01% Tween 20, following the maximum field application rate stated on the product label, i.e., 9.2 × 10^7^ cfu/L for *B. bassiana* and 1 × 10^10^ cfu/L for *A. muscarius*. The control treatment consisted of water with 0.01% Tween 20 only (indicated as CONT in the text).

The exact same APF and doses were applied in both the laboratory assays and the field trials.

### 2.2. Laboratory Assays: General Details

Laboratory assays were carried out in two different laboratories at the Department of Soil, Plant and Food Sciences (DiSSPA), UniBa, Bari, Italy, and at the Department of Agricultural and Forestry Sciences (DAFNE), UniTus, Viterbo, Italy. The same methodology was used in both laboratories in the same year.

Twigs with big buds were collected from the field during the winter of 2023–2024, the winter of 2024–2025 and the early spring of 2025 to set up the protocol and to investigate the potential APF efficacy across seasons. The infested galls were collected from the same commercial hazelnut orchard (cv. Tonda Gentile Romana) and conducted integrated pest management in Vitorchiano, district of Viterbo (lat. 42°27′41″ N, long. 12°11′14″ E), during the plant dormancy. The collected twigs were stored in sealed plastic bags at about 4 °C. They were used for the assay within two weeks of their collection. Before the application, the twigs were brought to room temperature, the bud galls were selected, taking only the intact ones, and surface-sterilized through immersion in 0.5% sodium hypochlorite solution for 10 s, rinsed in sterile water for 10 s and then left to dry under a flume cap.

At the end of the assays, a few mites were randomly removed from each treated organ, cleared, slide-mounted according to Keifer’s methods [20] and examined under a BX50 light microscope (Olympus, Tokyo, Japan) to gather evidence of the development of fungi into the mite body.

In addition, fungal re-isolations were performed to demonstrate the feasibility of re-isolating the fungi. For each treatment, 20 dead mites were randomly selected from at least four different replicates. They were exclusively transferred into the PDA medium and were incubated in the dark at 25 ± 1 °C. Each PDA Petri dish hosted between 4 and 8 individuals. All procedures were performed under sterile conditions (utilization of sterilized microneedles under the stereomicroscope for mite transferring, Bunsen burner constantly lit next to the microscope, etc.) to minimize external contamination.

### 2.3. Laboratory Assays: Protocol Applied in Winter 2023–2024

In the winter of 2023–2024, a first preliminary assay was conducted to design the most appropriate experimental protocol for the laboratory assays, based on those by Kanagaratnam et al. [17] and Abramishvili and Burjanadze [18].

**Figure 1:**
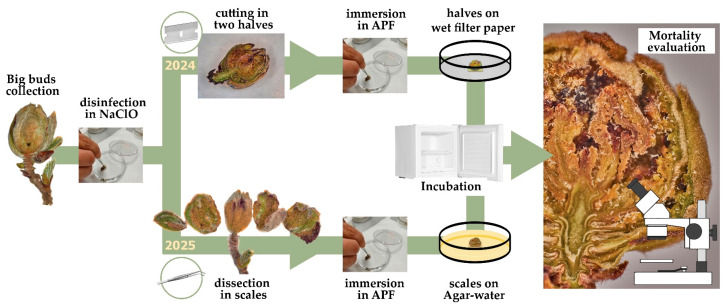
Laboratory protocol flowcharts: 2024 line regards trials carried out during winter 2023–2024; 2025 line regards trials carried out during winter 2024–2025 and early spring 2025.

Surface-sterilized bud galls were cut in two halves, checked under a stereomicroscope for the integrity of the tissues and presence of live eriophyoids, and immersed for 30 s into the conidial suspensions of NAT or MYC or into the CONT solution, following Midthassel et al. [21]. Then, the cut sides of the treated halved buds were individually placed up on a square of double-sided tape over a Whatman cellulose filter in 6 cm diameter Petri dishes and incubated in the dark at 25 ± 1 °C, with sterile water added every 1–2 days (Figure 1).

One week after the application, they were inspected under a dissecting microscope and the occurrence of live eriophyoids on the surface of the halved bud galls was evaluated on the total visible individuals in two classes: “halves with dead mites”, i.e., halves with totally visible eriophyoids, immotile or dead, or “halves with live mites”, i.e., halves with at least one or more live eriophyoids.

Mites were considered to be alive if they were motile or feeding, identified by subtle signs such as micromovements of legs, upright posture or leverage on the substrate; in cases of doubt, they were stimulated by an eyelash. Dead mites appeared rigid and immobile and eventually deteriorated. Color was not used as a criterion for distinguishing live and dead mites, due to the intraspecific variation which could be caused by feeding, ontological stage and age. In some cases, dead mites developed visible mycelial growth.

Sixteen replicates (each composed of a half bud) per treatment were planned for this year and for each laboratory.

### 2.4. Laboratory Assays: Protocol Applied in Winter 2024–2025 and Early Spring 2025

In the winter of 2024–2025 (at DiSSPA) and in the early spring of 2025 (at DAFNE), the protocol applied in the laboratory assay was slightly modified, considering the insights acquired in the previous year.

Scales were separated from the whole infested and surface-sterilized bud galls, using sterile tweezers, and these scales were used for the assays. This modification was necessary because the compact half of a bud gall does not allow a full wetting of all bud scales surfaces into the conidial suspensions, leaving untreated areas and mites. On the contrary, the single scale is much easier to manage for this scope. In addition, scales were easier to inspect, and the agar–water substrate allowed us to keep a more constant and suitable humidity during the experiment.

The bud gall scales were checked for the integrity of the tissues and the presence of live eriophyoids under a stereomicroscope, fully immersed into the conidial suspensions of NAT, MYC, or into CONT for 30 s. The treated bud scales were individually placed with the inner side up on sterile 1% water agar (Figure 1) in 6 cm diameter Petri dishes, incubated in the dark at 25 ± 1 °C, and inspected under a dissecting microscope after 24 h and 1 week after the application. At 24 h, fifteen mites were randomly selected from each scale and assessed individually as alive or dead (as reported above), while at the 1 week inspection, all mites found on the exposed surface of the scales were counted and classified accordingly. The mortality rate was calculated as the number of dead eriophyoids on the total number of counted mites on the treated bud scale.

Thirty replicates per treatment were planned for the assays of this year and for each laboratory.

### 2.5. Field Trials

Following the preliminary data collected in the laboratory during winter 2023–2024, two field assays were carried out in the commercial orchard of Vitorchiano, from which bud galls were collected for the laboratory assays. Site climatic parameters, namely air temperature (°C) and relative humidity (as percentage), were continuously recorded using an xSense mini weather station (xFarm Technologies Italia srl, Milan, Italy) (Tables S1 and S2; Figures S1 and S2).

**Figure 2:**
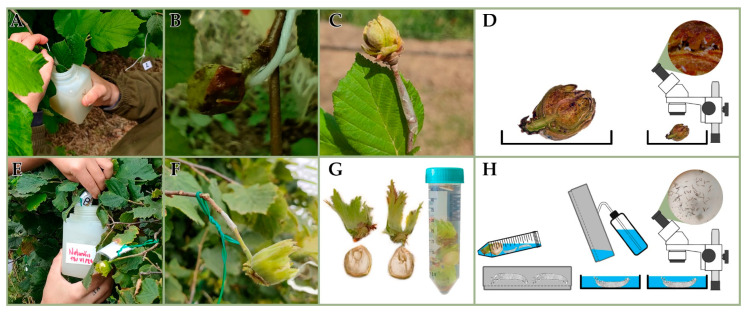
Field trials conducted in 2024: (**A**) big buds immersed in conidial suspensions; (**B**) wet big buds retaining conidial suspension after APF application; (**C**) a strip of double-sided adhesive tape used as a sticky trap; (**D**) inspection of buds and tapes under a dissecting microscope for the mortality assessment; (**E**) nuts immersed in conidial suspensions; (**F**) a strip of double-sided adhesive tape placed at the base of the nut insertion; (**G**) nuts collected individually and washed in a 50 mL centrifuge tube on an oscillating laboratory shaker; (**H**) nuts washed to dislodge mites on their surface, filtered through a 500 mesh sieve (gray rectangle) to recover the mites, subsequently poured in a 9 cm diameter Petri dish and counted under a dissecting microscope.

A first trial (Figure 2) was conducted during the late big bud break in 2024 (BBCH 19-30, according to Taghavi et al. [22]), while the mite migration was still in progress. A double-sided tape, 5 mm wide, was applied at the base of the insertion of 40 bud galls and played as a sticky trap for mites emerging from the galls after the treatment (Figure 2C). Soon after, all selected bud galls were dipped on the same day in the conidia suspension of NAT (n = 20) and treated with CONT (n = 20) as a control (Figure 2A,B). MYC was not applied, due to the poor results of the laboratory experiments conducted during the winter 2023–2024 on the gall-making species. No big buds were damaged during the treatments and they remained healthy on the twigs. Fourteen days after the APF application, all galls (NAT and CONT) and tapes were detached from the twigs, separately collected and examined in the laboratory under a dissecting microscope to evaluate live and dead mites (Figure 2D). During the first field trial, the mean daily temperature was 17.7 ± 0.17 °C, and the mean relative humidity was 71.9 ± 2.57% (Table S1; Figure S1). Each big bud was cut in two halves, and their cut surfaces were inspected, assigning one of the four mortality classes (I = 0; II = 0.1–33.3% dead mites; III = 33.4–66.6% dead mites; or IV = 66.7–100% dead mites) to each bud in a sample of at least 25–30 individuals/buds that are randomly selected under the observation field among a larger population. The identification of the dead/live state was carried out according to the same criteria used in the laboratory assays. None of the big buds were found without mites.

A second trial (Figure 2) was carried out during nut development in 2024 at BBCH 79 (according to Taghavi et al. [22]). Single nuts on twigs were dipped on the same day in a conidia suspension of NAT (n = 20), MYC (n = 20) and CONT (n = 20) (Figure 2E). Both species of APF were assayed, based on the suspicion that the population on the nuts should be composed mainly of the free-living species of *P. avellanae* and not by the gall-making one. Double nuts were handled, keeping only one nut on the same twig insertion before APF application. A double-sided tape, 5 mm wide, was tied at the base of the insertion of nuts and played as a sticky trap for mites coming out from the nuts after the treatment (Figure 2F). No nuts were damaged during the treatments and they remained healthy on the twigs. During the second field trial, the mean daily temperature was 22.1 ± 0.85 °C, and the mean relative humidity was 64.1 ± 2.32% (Table S2; Figure S2). Fourteen days after the application, all nuts (NAT, MYC and CONT) and tapes were detached from the twigs, separately collected and transported to the laboratory (Figure 2G). Tapes were directly examined under a dissecting microscope. Nuts were submitted to the washing and sieving technique [23] for mite extraction, modified for massive washing on an oscillating laboratory shaker for 10 min 40 RPM (Figure 2G,H), then mites were counted under a dissecting microscope.

### 2.6. Statistical Analysis

Statistical analyses were carried out in RStudio (version 2023.12.0 + 369). Linear and generalized linear mixed effects models were used to explore the effect of APF applications on the *P. avellanae* mortality rate. For a simple graphical interpretation of the results, the graphs and results displayed the mortality percentage (i.e., the mortality rate multiplied by 100) (see below). Two general linear mixed-effects models were hence run to test the effect of APF on the eriophyoid mortality in the laboratory assays carried out during winter 2024–2025 (big bud dormancy) at DiSSPA (Model 1) and spring 2025 (big buds spreading) at DAFNE (Model 2), over the time. Treatment (categorical; three levels), time (categorical; two levels) and the interaction between treatment and time were used as predictors.

Additionally, two linear models were run to test the effect of APF applications on the mortality of eriophyoids in the field assay on the bud galls (Model 3) and on the abundance of eriophyoids in the field assay on the nuts (Model 4). Treatment (categorical) was used as a predictor. The abundance of eriophyoids was sqrt-transformed to meet assumptions for linear models. For all the models, sterile water (i.e., control treatment) was set as the baseline.

Models were checked using the “DHARMa” package [24] for overdispersion and residual distribution. The significance of study factors was then assessed using the ‘ANOVA’ function from the ‘car’ package [25]. Finally, Tukey’s multiple comparison test was applied to determine the significance among treatments.

## 3. Results

### 3.1. Laboratory Assays: Protocol Applied in Winter 2023–2024

Laboratory results indicated differences between control treatments and those with APF. In the two laboratory trials, all big bud halves (16/16; 100%) treated with NAT and MYC resulted in complete mite mortality 1 week after the treatment. Regrettably, in 9 out of 16 and in 11 out of 16 big bud halves of CONT in DAFNE and DiSSPA, respectively, the observed mites were dead or immotile, whereas the remaining buds showed signs of mite viability. The high mortality observed in the CONT treatment hence suggested that the applied protocol may present several methodological issues, requiring adjustments to be implemented in the following year (i.e., disinfection of treated tissue, constant humidity).

**Figure 3:**
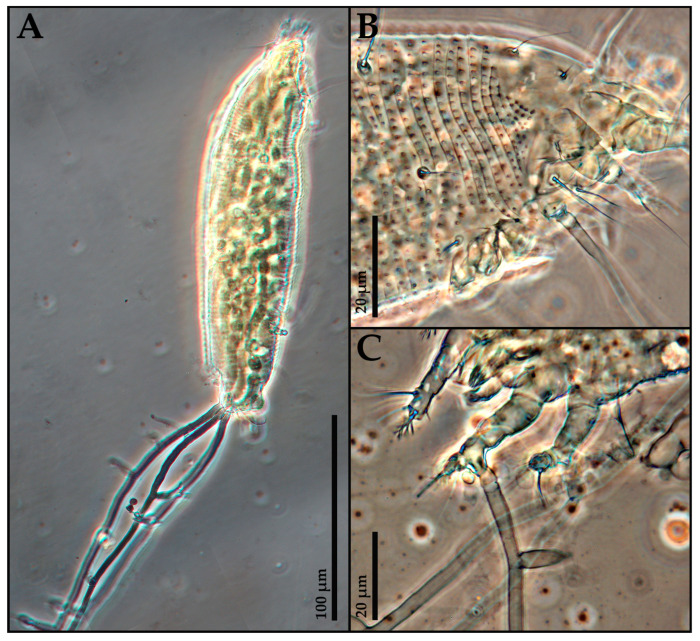
Light micrographs of *Phytoptus avellanae* mites after 1 week of exposure to NAT and infected by *Beauveria bassiana* from the assays carried out at DiSSPA laboratory: (**A**) lateral view of a specimen with the fungal mycelium emerged from the anal lobes; (**B**) lateral view of the anterior part of the body of a specimen with hyphae emerging from the coxal area; (**C**) ventral view of a specimen with hyphae emerging from the first leg.

A microscopic examination of clarified specimens confirmed fungal development exclusively in mites exposed to *B. bassiana*. Fungal hyphae were observed both within the mite body and emerging from its surface, with the formation of fruiting (Figure 3).

Only the NAT treatment yielded positive re-isolation results. In fact, *B. bassiana* was successfully re-isolated in the DAFNE (3 mites out of 20) and DiSSPA (10 mites out of 20) laboratories after transferring the infected mites, whereas the remaining attempts did not produce fungal colonies.

### 3.2. Laboratory Assays: Protocol Applied in Winter 2024–2025 and Early Spring 2025

**Figure 4:**
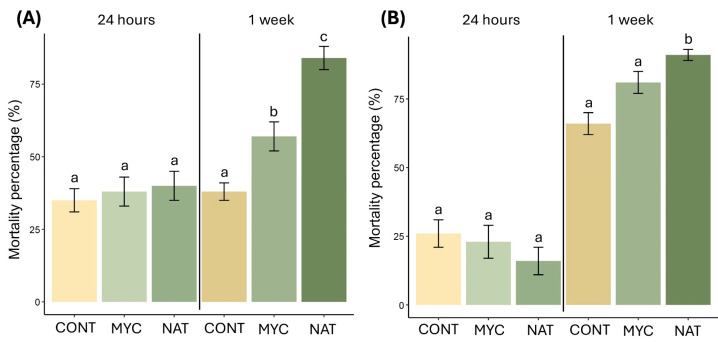
Mortality percentage (means ± SE) of *Phytoptus avellanae* 24 h and 1 week after the treatment at DiSSPA (**A**) with bud galls collected in winter 2024–2025 and DAFNE laboratories (**B**) with bud galls collected in spring 2025. Letters on bars indicate the results of Tukey’s pairwise comparison test (*p* < 0.05); different letters indicate statistically significant differences between treatments. Treatment abbreviations: CONT: water plus Tween 20; MYC: Mycotal^®^; NAT: Naturalis^®^.

At DiSSPA, we found no significant differences between treatment after 24 h, resulting in a mortality of 41.7 ± 5.5% and 39.2 ± 5.6%, respectively, for NAT and MYC, compared to 35.7 ± 4.2 of the CONT (Figure 4A). A significant difference was pointed out 1 week after the application, with the NAT treatment causing higher mean mortality (87.3 ± 3.2%) than MYC (57.1 ± 5.5%) and CONT (38.1 ± 3.5%) (Figure 4A, Table 1).

At DAFNE, despite the difference in the age of the treated bud galls age, we found similar results compared to those obtained at the DiSSPA laboratory. The mean mortality 24 h after the application did not show any difference between the treatments (Figure 4B). The mean mortality at 1 week after the application showed a significantly higher mortality of bud gall scales treated with the NAT (91.0 ± 2.7%), MYC (81.1 ± 4.0%) and CONT (66.7 ± 4.6%) treatments (Figure 4B, Table 1).

**Figure 5:**
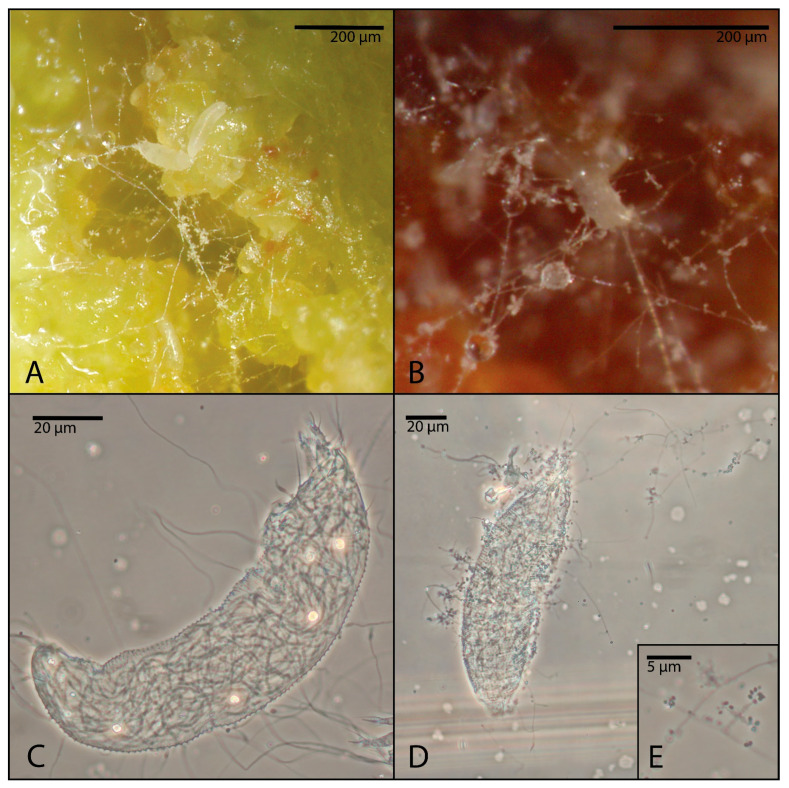
Light micrographs of *Phytoptus avellanae* mites after 1 week of exposure to NAT and infected by *Beauveria bassiana* from the assays carried out at DAFNE laboratory: (**A**,**B**) specimens covered by the fungal mycelium emerging from the body; (**C**,**D**) specimens with fungal hyphae within the body and emerging from it (**C**,**D**); (**E**) isolated *B. bassiana* conidiophores branches, cultured through acarine isolation on PDA.

Microscope observations on treated galls and mounted specimens confirmed the presence of *B. bassiana* mycelium within the mite body (Figure 5).

During galls’ incubation, the fungus was able to epiphytically develop, extending externally from the infected mite’s body (Figure 5A,B). Confirmation of internal colonization within the mite was obtained by focusing on median sections under the microscope, where numerous fungal hyphae were observed colonizing the body of the dead mites and emerging as outgrowing hyphae. These structures subsequently produced conidiophore branches that are typical of *B. bassiana* (Figure 5C–E).

PDA re-isolations performed in the 2024–2025 assays resulted in successful recovery of the same fungal strain inoculated during the assays only in the case of *B. bassiana* (Figure 5E): 12 out of 20 at DiSSPA and 8 out of 20 at DAFNE. The remaining re-isolations yielded either contaminant bacterial/fungal growth or no cultures at all.

### 3.3. Field Trials

**Figure 6:**
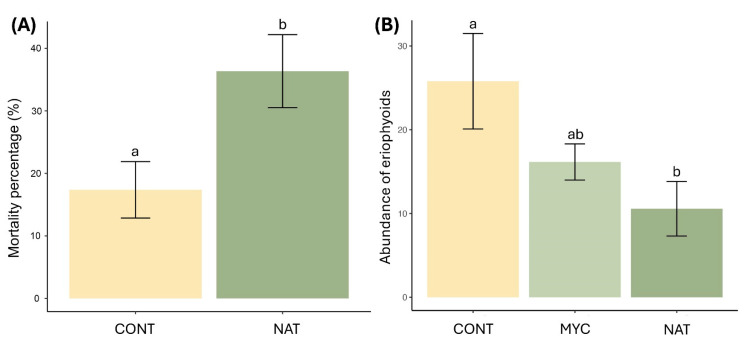
Effects of the application of APF on *Phytoptus avellanae* in the field trials: (**A**) mortality class (means ± SE) of treatments on bud galls; (**B**) mite abundance after the treatment on nuts. Abundance values were sqrt-transformed. Letters on bars indicate the results of Tukey’s pairwise comparison test (*p* < 0.05); different letters indicate statistically significant differences between treatments. Treatment abbreviations: CONT: water plus Tween 20; MYC: Mycotal^®^; NAT: Naturalis^®^.

Field trials on bud galls and nuts exhibited significant differences between treated and not treated plant organs (Table 2). Bud galls immersed in NAT recorded a higher mortality compared to the CONT (Figure 6A). Moreover, sticky tapes placed at the base of the big buds did not catch mites escaping from the galls treated with NAT. Vice versa, tape mounted on the CONT treatment captured an average of 21.8 ± 4.0 mites, demonstrating an active migration till at the time of this trial.

The counting of mites after the nut treatment was also significantly lower in the nuts immersed in NAT (10.6 ± 3.2 mites) compared to nuts treated differently (CONT 25.8 ± 5.8 mites, MYC 16.2 ± 2.2 mites) (Figure 6B). No individuals were captured escaping from all treated nuts (late June 2024).

## 4. Discussion and Conclusions

The study had two aims. The first was to develop a detailed laboratory and field protocol for evaluating the efficacy of APF against gall-making eriophyoid mites, verifying its suitability and challenges and identifying possible shortcomings. The second relied on using the novel protocol to assess the potential efficacy of selected commercial APF against *P. avellanae*. The present two years of experimentation in the laboratory and field settings were able to generate data and information concerning the appropriateness of the applied protocols, improving the procedures that were previously applied against another gall-making eriophyoid species (*C. ribis*) [17] and the same *P. avellanae* [18]. The scarcity of the literature on this issue involving gall-marking eriophyoid mites [5] does not allow for a large comparison with previous data.

*Phytoptus avellanae* is well known as a gall-making mite on hazelnut, causing negative yield effects in nuts. The impact of the free-living and cryptic entity under the *P. avellanae sensu lato* name has not been already assessed. The biological characteristic of inducing and inhabiting galls makes *P. avellanae sensu stricto* substantially more challenging to target compared to non-gall-making (i.e., free-living) mites. In hazelnut, yield losses caused by this gall mite result from its early infestation of newly formed mixed buds, which prevent these buds from entering production. The big buds infested by *P. avellanae* represent an almost closed and inaccessible structure, making them far less amenable to conventional control strategies, apart from the migration period. Free-living mites typically exert their damaging activity on exposed plant organs such as leaves, where populations can be readily monitored and undergo a treatment, and they are also more suitable for laboratory rearing on experimental arenas.

In the laboratory assays carried out during winter 2023–2024, both APF formulations caused 100% mortality after 1 week from the treatment, providing initial evidence for supporting the potential efficacy of the applied commercial APF strains. However, the high mortality recorded in the control suggested that these findings should only be considered preliminary and are not suitable for a robust statistical analysis. The experimental protocol evidenced the need for improvements and refinements. Specifically, a fluctuation in the substrate moisture was observed, alternating between excessive hydration and desiccation during the day. In the subsequent assays, water agar substrate appeared to be able to preserve the plant’s material humidity and avoid periodical addition of water to the substrate, which could have also been a cause of contamination. In addition, the halves of the big buds appeared to be structurally compact, which hindered both uniform treatment applications and accurate quantification of the efficacy of the treatment. In fact, mites can move among the scales, reach deeper spaces and remain hidden from the operator inspections. In the subsequent assays, single scales were used and better treated. The mites had fewer opportunities to hide, and counting was easier.

The laboratory assays carried out in winter 2024–2025 and early spring 2025 allowed us to correct the previously applied protocol and account for the mite biology. In fact, it was observed that galls collected in winter and in early spring had a different population structure, with more adults in winter, and more juveniles and eggs in the early spring, as expected. Consequently, the experiments conducted in the DISSPA and DAFNE laboratories could not be regarded as replicates, but rather as separated assays performed on different biological/physiological stages of the mite population within the galls. This approach aimed to address whether the population structure could influence the treatment efficacy, due to the presence of stages that were potentially more or less susceptible to APF infection.

Statistical analyses revealed that both treatment and time significantly affected eriophyoid mortality in the laboratory assay that was carried out in winter 2024–2025. It is quite interesting to observe that the formulation based on *B. bassiana* exhibited almost the same higher efficacy (more than 85% of mean mortality) on both experimental conditions, whereas the formulation based on *A. muscarius* appears to be much more efficient when applied on galls collected in the early spring. However, the higher mite mortality on the galls treated just with water and Tween 20 for the galls collected in early spring should be considered, with respect to those collected in winter 2024–2025. These data can show a certain susceptibility of the mites to the environmental conditions, owing to the need to go out from the buds and look for a new bud.

The first field trial was planned to simulate a canopy treatment aimed at maximizing wetting of the target organs as closely as possible, i.e., when the big buds were opening for leaving mites of *P. avellanae sensu stricto* to migrate. In the field assays, the results showed higher mortality in the big buds treated with the formulation based on *B. bassiana*. The efficacy of the treatment at this BBCH was further confirmed by the data from the sticky tapes placed at the base of the big buds, which were null for the treated big buds and positive for the control. In this context, the potential epiphytic and endophytic colonization of *B. bassiana* [26], also on the bud galls, may have further favored the success of the application.

The second field trial was planned to mainly contrast the free-living species, which was supposed to be much more present on the vegetation during the development of the nuts. The results showed a significant reduction in eriophyoid abundance on the nuts, indicating the efficacy of the treatment under field conditions. The fact that mites were not also captured on sticky traps at the nut insertion for the control could be considered a normal event, because these mites prefer to maintain their population on the apical parts of the plant (Ozman-Sullivan S., pers. comm.).

The use of plant protection products is becoming increasingly restricted due to the need for more sustainable production systems, and hazelnut cultivation is not an exception. In Europe, the management of the hazelnut gall mite has traditionally relied on few chemicals and biopesticides. Hazelnut production almost completely lacks studies on the use of APF as a tool for plant protection against mite pests. The findings of this study carried out in the laboratory and field may provide a basis for developing a novel management strategy for hazelnuts, offering alternative means. In fact, the experimental results support further exploration of field application approaches for the control of *P. avellanae* in hazelnut orchards. The evaluation of the efficacy of a strain of *A. muscarius* found on the hazelnut galls and currently under study [10] could be the next step.

Considering the ecological context, a spring application of the APF might appear to be more rational for the control of *P. avellanae*, as temperatures and solar radiation are generally lower and relative humidity is higher than in the summer. It remains uncertain whether the gall-making species may be more susceptible to fungal penetration of the cuticle than the free-living species. The gall-making individuals are adapted to the relatively buffered environment of the gall, rather than the harsher conditions of the phylloplane. This adaptation might have a dual effect. On the one hand, the gall constitutes a closed microhabitat that shields mites from external agents, representing a physical barrier that fungal spores may find difficult to overcome. This limitation is evident when APF are applied during the overwintering phase, when mites remain enclosed within galls. Unlike sulfur, which acts only through direct contact with exposed individuals, APF conidia, like those of *B. bassiana*, may adhere to the mites’ body, travel with it to the new buds where new galls will form next in the year, and, subsequently, colonize the bud through their epiphytic growth capacity or by a horizontal transfer among mites via direct contact [27].

## Figures and Tables

**Table 1:** Results of the generalized linear mixed models testing the effect of APF on *Phytoptus avellanae* in the laboratory assays.

Variable	Chisq	*p*-Value
Model 1: Mortality rate of eriophyoids at DiSSPA laboratory		
Treatment	36.43	<0.001
Time	34.80	<0.001
Treatment × Time	22.29	<0.001
Model 2: Mortality rate of eriophyoids at DAFNE laboratory		
Treatment	2.06	0.472
Time	185.34	<0.001
Treatment × Time	11.12	0.003

**Table 2:** Results of the linear mixed models testing the effect of APF on *Phytoptus avellanae* in field trials.

	Sum of Square	F Value	*p*-Value
Model 3: Mortality class of eriophyoids in the field assay on bud galls			
Treatment	3.20	6.40	0.016
Model 4: Abundance of eriophyoids in the field assay on nuts			
Treatment	47.31	6.02	0.004

## Data Availability

The original contributions presented in this study are included in the article/Supplementary Material. Further inquiries can be directed to the corresponding author.

## Supplementary Materials

The following supporting information can be downloaded at: https://www.mdpi.com/article/10.3390/insects16111182/s1. Table S1: Daily Trend of Temperature and Humidity during first field trials, starting on 10 May 2024–ending on 24 May 2024. Figure S1: Graph of daily Trend of Temperature and Humidity during first field trials, starting on 10 May 2024–ending on 24 May 2024. Table S2: Daily Trend of Temperature and Humidity during second field trials, starting on 13 June 2024–ending on 27 June 2025. Figure S2: Graph of d aily Trend of Temperature and Humidity during second field trials, starting on 13 June 2024–ending on 27 June 2025.
