# Supplementary material for: Laboratory and Field Bioassays of Arthropod Pathogenic Fungi Application for the Control of the Hazelnut Big Bud Mite, Phytoptus avellanae s. l
Source: Insects. 2025 Nov 20;16(11):1182. doi: 10.3390/insects16111182 (PMC12653174; doi:10.3390/insects16111182)
Supplement: Supplementary file 1 [file insects-16-01182-s001.zip › insects-3900573-supplementary.pdf]

Supplementary material

Table S1: Daily Trend of Temperature and Humidity during first field trials, starting on 10th May 2024 – ending on 24th May 2024

Date	Aver. Temp (°C)	Max temp. (°C)	min temp (°C)	Aver. Rel. humidity (%)
10-May	18.0	24.3	11.4	57.0
11-May	18.1	24.9	10.7	55.0
12-May	18.0	25.0	11.3	60.7
13-May	17.3	24.2	11.0	66.9
14-May	18.4	25.6	10.9	67.4
15-May	18.5	26.6	12.4	75.8
16-May	18.5	26.2	13.2	71.7
17-May	17.7	24.1	9.7	64.1
18-May	17.3	25.0	9.9	68.8
19-May	17.8	22.0	12.7	79.1
20-May	18.4	23.9	13.2	82.9
21-May	17.9	22.6	13.2	80.8
22-May	16.3	22.0	11.4	87.1
23-May	16.7	23.2	10.4	80.5
24-May	17.1	24.6	10.6	80.0

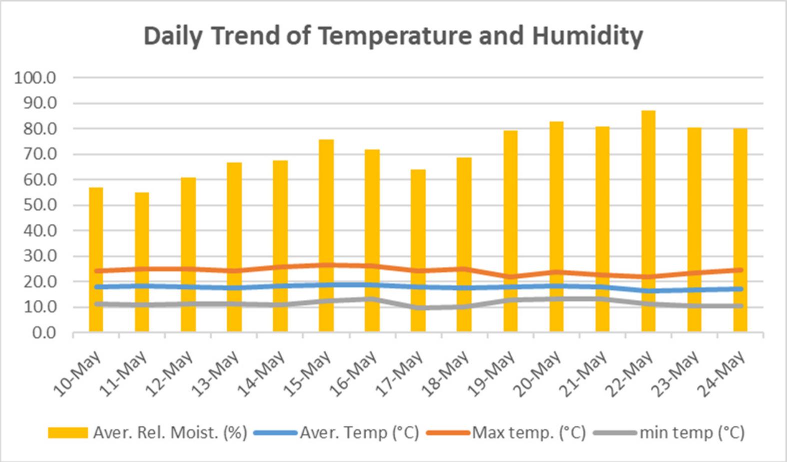

Figure S1: Graph of daily Trend of Temperature and Humidity during first field trials, starting on 10th May 2024 – ending on 24th May 2024

Table S2: Daily Trend of Temperature and Humidity during second field trials, starting on 13th June 2024 – ending on 27th June 2025

Date	Aver. Temp (°C)	Max temp. (°C)	min temp (°C)	Aver. Rel. Moist. (%)
13-Jun	16.7	22.3	12.0	78.4
14-Jun	19.2	25.8	12.2	65.9
15-Jun	20.4	27.6	11.2	66.3
16-Jun	20.7	27.8	13.4	69.3
17-Jun	22.8	30.5	15.0	62.3
18-Jun	25.0	33.2	16.5	58.9
19-Jun	25.0	32.0	18.4	52.9
20-Jun	28.0	36.5	20.7	47.5
21-Jun	27.3	36.1	21.3	53.9
22-Jun	24.4	30.4	18.1	55.0
23-Jun	19.7	25.3	14.6	68.1
24-Jun	18.4	25.5	13.2	76.6
25-Jun	19.9	25.7	12.9	72.7
26-Jun	20.6	25.9	14.2	69.8
27-Jun	22.9	29.1	15.4	64.4

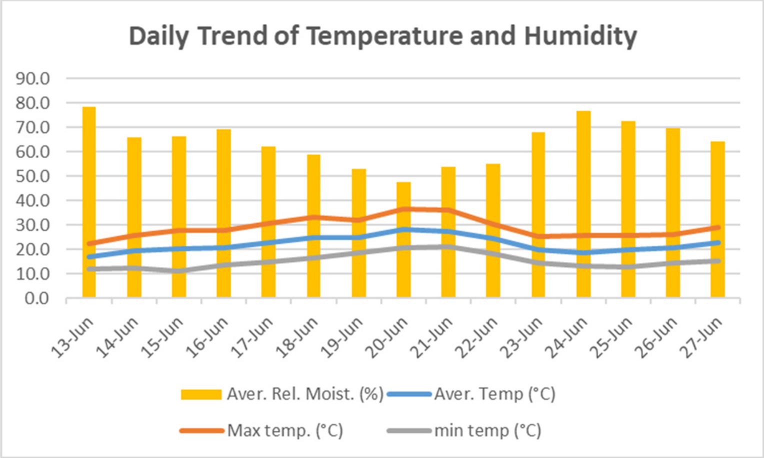

Figure S2: Graph of d aily Trend of Temperature and Humidity during second field trials, starting on 13th June 2024 – ending on 27th June 2025
